# MET/PKCß expression correlate with metastasis and inhibition is synergistic in lung cancer

**DOI:** 10.4103/1477-3163.57857

**Published:** 2009-11-24

**Authors:** Leonardo Faoro, Gustavo M. Cervantes, Benjamin D. Ferguson, Tanguy Y. Seiwert, Soheil Yala, Wicki T. Vigneswaran, Maria Westerhoff, Maria S. Tretiakova, Mark K. Ferguson, Glaci L. Moura, Aliya N. Husain, Everett E. Vokes, Ravi Salgia

**Affiliations:** Section of Hematology/Oncology, Department of Medicine, University of Chicago Pritzker School of Medicine, and University of Chicago Cancer Research Center, Chicago, IL 60637, USA; 1 Section of Hematology/Oncology, Department of Surgery, University of Chicago Pritzker School of Medicine, and University of Chicago Cancer Research Center, Chicago, IL 60637, USA; 2 Section of Hematology/Oncology, Department of Pathology, University of Chicago Pritzker School of Medicine, and University of Chicago Cancer Research Center, Chicago, IL 60637, USA; 3 Department of Hematology/Oncology, Hospital de Clínicas, Universidade Federal do Paraná, Curitiba, Brazil

**Keywords:** c-MET, developmental therapeutics, lung cancer, protein kinase C

## Abstract

**Background:**

Treatment of non-small cell lung cancer (NSCLC) remains a difficult task in oncology. Targeted inhibition of oncogenic proteins is promising. In this study, we evaluate the expression of MET and PKCß and in vitro effects of their inhibition using SU11274 and enzastaurin (LY317615.HCl) respectively.

**Materials and Methods:**

Patient samples were analyzed by immunohistochemistry for expression of PKCß and MET, utilizing tissue microarrays under an IRB-approved protocol. Expression of PKCß and MET was evaluated in cell lines by immunoblotting. Treatment with SU1174 against MET and enzastaurin against PKCß was performed in H1993 and H358 cell lines, and cell proliferation and downstream signaling (phosphorylation of MET, AKT, FAK, and GSK3ß) were evaluated by immunoblotting. Statistical analysis was performed using SPSS 16.0.

**Results:**

Expression of MET positively correlated with lymph node metastases (p=.0004), whereas PKCß showed no correlation (p=0.204). MET and PKCß expression were also strongly correlated (p<0.001). Expression of MET was observed in 5/8 cell lines (H358, H1703, A549, H1993, H2170; absent from H522, H661, or SW1573), whereas PKCß expression was observed in 8/8 cell lines. Cell proliferation was significantly impaired by treatment with SU11274 and enzastaurin, and their effects were synergistic in combination (CI=0.32 and 0.09). Phosphorylation of MET, FAK, AKT, and GSK3ß were strongly inhibited with both agents in combination.

**Conclusions:**

Concomitant inhibition of MET and PKCß significantly increased cytotoxicity in vitro against NSCLC, disrupting important downstream signaling pathways. Further evaluation in animal models is warranted.

## BACKGROUND

Lung cancer is the most common cause of cancer mortality in the United States and worldwide.[1,2] The disease presents commonly in advanced stages, which renders prognosis poor.[3] Non-small cell lung cancer (NSCLC) is the most common form of lung cancer, accounting for approximately 80% of lung cancer cases. Though multidisciplinary therapeutic approaches toward NSCLC have improved survival and morbidity, the survival rates for advanced NSCLC remains dismal even with novel chemotherapy.[4] Inhibitors against epidermal growth factor receptor (EGFR) tyrosine kinase and antibodies against the ligand-binding domains of EGFR (such as cetuximab) that have come to clinical fruition produce only modest improvements in clinical outcomes.[5] However, there are multiple other molecular abnormalities in lung cancer yet unexplored.[6]

The protein kinase C (PKC) family of serine-threonine protein kinases has been implicated in several important cellular functions including proliferation, motility, invasion, and apoptosis.[7] Of the various PKC isoforms, PKCß expression has been demonstrated in several human cancers, most notably B cell lymphomas.[8] Its overexpression has been shown to be an adverse prognostic factor in diffuse large B cell lymphomas.[8–10] This was evaluated in a gene expression study, where 6817 genes were evaluated in relation to refractoriness versus curability in diffuse large B cell lymphomas; patients whose tumors had higher expression of PKCß2 had worse five-year event-free survivals (36 vs. 49%, p=0.054).[8] PKCß has been implicated in angiogenesis, making it an attractive target for therapeutic inhibition in cancer.[11] Downstream, PKC can target the PI3K/AKT pathway and other signal transduction pathways.[12,13] Enzastaurin (LY317615.HCl) is an oral small-molecule acyclic bisindolylmaleimide inhibitor of PKCß currently undergoing phase I-III clinical trials that inhibits PKCß in the low nanomolar range. At higher dosages, it may inhibit other PKC isoforms, most notably PKC alpha. It is currently being studied in multiple myeloma,[14] breast cancer,[15] cutaneous T-cell lymphoma,[16] thyroid cancer,[17] colon cancer, glioblastoma,[12] and non-small cell lung cancer.[18]

The c-MET receptor tyrosine kinase (MET) was originally identified as the cellular homologue of the TPR-MET oncoprotein.[19] MET is over-expressed in a number of malignancies, sometimes mutated (germline mutations/single nucleotide polymorphisms (SNPs) or somatic mutations), and at other times amplified. It has been shown to be a promising target in the treatment of cancer, with multiple compounds in pre-clinical and clinical development.[20]

Combination therapies, especially those involving MET inhibition, have been increasingly studied as a treatment strategy in lung cancer.[21–24] Given the current knowledge of roles of MET, a growth-factor receptor, PKCß, an intracellular target in lung cancer and the efficacy of their inhibition as independent targets, we have investigated whether their dual inhibition leads to increased cytotoxicity against NSCLC *in vitro*. Intracellular signaling networks were also evaluated for effects of the combination therapies.

## MATERIALS AND METHODS

### Tissue microarray and immunohistochemistry

Paraffin-embedded, formalin-fixed tumor tissues from patients with NSCLC with associated clinical information (histological subtype, clinical stage, overall survival and vital status) were processed into a tissue microarray (TMA) under an Institutional Review Board-approved protocol (UCMC #9571 and #13473). Immunohistochemistry (IHC) was performed using biotin-free HRP-labeled polymer complex bound to secondary antibody (DAKO Cytomation, Carpinteria, CA), and performed according to previously published procedures.[25] Negative controls were performed by substituting the primary antibody step with non-immune mouse immunoglobulins. Results were assessed semi-quantitatively (by three independent pathologists, MW/MT/AH) by light microscopy, and graded as negative (0), weak (1+), strong (2+), and very strong expression (3+). Sixty-nine patients were analyzed.

### Cell lines and culture

NCI-H522 (adenocarcinoma), NCI-H1703 (adenocarcinoma), A549 (adenosquamous carcinoma), NCI-H1993 (adenocarcinoma), NCI-H2170 (squamous cell carcinoma), NCI-H661 (large cell carcinoma), SW1573 (bronchoalveolar carcinoma) and NCI-H358 (bronchoalveolar carcinoma) cell lines were obtained from the American Type Culture Collection (Rockville, MD) All cell lines were cultured as per our established protocols.[26]

### Reagents and antibodies

Enzastaurin was provided by Eli Lilly (Indianapolis, IN). Fetal bovine serum (FBS) was obtained from Gemini Bioproducts (Woodland, CA). SU11274 was obtained from EMD Biosciences (San Diego, CA). Cell culture media, penicillin, and streptomycin were obtained from Cellgro (Boehringer Ingelheim, Heidelberg, Germany). Antibodies used included: anti-PKCß2, anti-Met, anti-phospho-FAK (Tyr925), anti-FAK, anti-phospho-AKT (Ser473) and anti-phospho-GSK3ß (Ser9) (Santa Cruz, Santa Cruz, CA); anti-phospho-Met (Tyr130/1234/1235) (Invitrogen, Carlsbad, CA); ß-actin monoclonal antibody (Sigma, St. Louis, MO). All other chemicals were purchased from Sigma (St. Louis, MO).

### Immunoblotting

To examine protein expression in NSCLC cell lines under basal conditions, subconfluent cells were cultured in medium supplemented with 10% FBS. To detect the inhibition of cell transduction pathways, cells grown on 10-cm culture dishes for 24 hours were washed twice with PBS and incubated at 37°C with 2.5 *μ*M enzastaurin, 2.5 *μ*M SU11274, or enzastaurin and SU11274 in combination (or DMSO as a negative control) for different durations as described, in serum-free media. Whole cell lysates were collected and immunoblotting was performed following routine protocols.[26] The same membranes were subsequently stripped and reprobed in a similar fashion with different primary antibodies. ß-actin levels were used to control for equal loading amounts.

### Cell proliferation studies

Cells were plated in 96-well plates at 5×10^3 cells per well in serum-containing media and grown for 24 hours. Drugs (or drug carrier) were added in serum-free media, and cells were incubated for 72 hours. Cell growth was estimated utilizing fluorometric readings after the addition of Alamar Blue (Invitrogen, Carlsbad, CA), a non-radioactive, non-toxic dye that is reduced and whose fluorescence is proportional to cellular metabolic activity. A HT Synergy Plus microplate reader (Biotek, Winooski, VT) was used to measure fluorescence (530-560 nm excitation wavelength and 590 nm emission). Drug synergism was estimated by the median-effect analysis[27] using the Calcusyn 3.0 software package (Biosoft, Camdridge, UK).

### Statistical analysis

Comparisons between average means of protein expression were made by utilizing the Student's t-test for independent samples. For evaluation of differences in frequencies among different categories, the Chi-square test was used. The statistical software used was SPSS, version 16.0 (SPSS Inc, Chicago, IL).

## RESULTS

### Expression and correlation of PKCß and MET in NSCLC tumor samples

**Figure 1:**
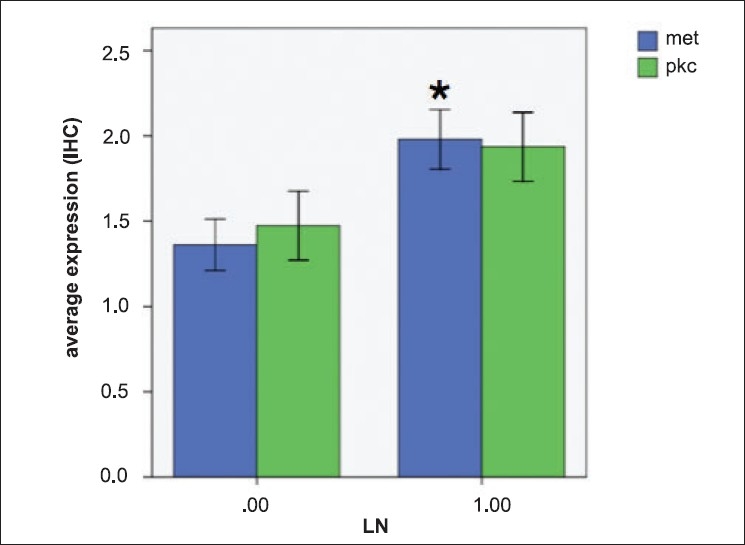
Expression of PKCß and MET in NSCLC tumor tissues. A total of 62 patient samples are evaluated with immunohistochemistry for PKCß and MET expression, and patients were grouped by the status of lymph node (LN) metastasis (No = negative metastasis to LN, n=35; Yes= positive metastasis to LN, n=27). Expression of MET in LN-positive patients was significantly higher than LN-negative patients (blue bars, p=0.009), whereas PKC expression revealed no significant difference (green bars, p=0.114). P values are evaluated by Student's T test.

Evaluation of protein expression of PKCß and MET in the TMAs was performed. Table 1 lists the characteristics of the 69 patients included in the analysis. Most patients were older individuals, with a slight male predominance. Forty-five per cent of patients had adenocarcinomas, followed by large cell carcinomas (23%) and squamous cell carcinomas (14%). Given that most samples were derived from curative lung resections, 55% of patients had clinical stage I disease. The expression of PKCß and MET among patients whose tumors had not metastasized to lymph nodes (clinical stage I) versus those whose had (stages II-IV) was compared [Table 1]. MET expression was significantly increased in patients with positive lymph nodes (1.97 versus 1.36, p=0.009, Student's t-test). PKCß expression tended to be also higher in patients with positive lymph nodes; however, statistical significance was borderline (1.93 versus 1.47, p=0.114, Student's t-test) [Figure 1]. In order to further evaluate this, we also grouped patients according to whether they had strong versus weak expression of PKCß and MET and correlated these two variables [Table 2]. Again, MET expression positively correlated with the presence of positive lymph node metastasis (p=0.004, Chi-square), while PKCß expression was not significantly associated with lymph node metastasis (p=0.204). The association between MET and PKCß expression was also investigated in the same fashion. There was a strong positive correlation between PKCß and MET expression in the NSCLC samples (p<0.001, Chi-square) [Table 2].

**Table 1:** Characteristics of 69 Patients whose Samples were Included in the TMA Analysis

Age at diagnosis (years)	63.5 (+/− 10.4)
Gender	
Male	40 (57.9%)
Female	29 (42.1%)
Histology	
Adenocarcinoma	31 (44.9%)
Squamous cell carcinoma	10 (14.4%)
Large cell carcinoma	16 (23.1%)
NSCLC	6 (8.6%)
BAC	4 (5.7%)
Mixed	1 (1.4%)
Other	1 (1.4%)
Clinical Stage	
I	38 (55%)
II	9 (13%)
III	18 (26%)
IV	4 (6%)

**Table 2:** Correlation of MET and PKC expression in 69 patient samples

	LN neg	LN pos	*P*=0.004
MET Low	23	8	
MET High	15	23	
PKC Low	18	10	*P*=0.204
PKC High	20	21	
	MET Low	MET High	*P*<0.001
PKC Low	20	8	
PKC High	11	30	

According to LN Status and between each other. Low ≤ average, high > average expression. Statistical significance calculated using Chi-square test.

### MET and PKCß Expression in NSCLC Cell Lines:

**Figure 2:**
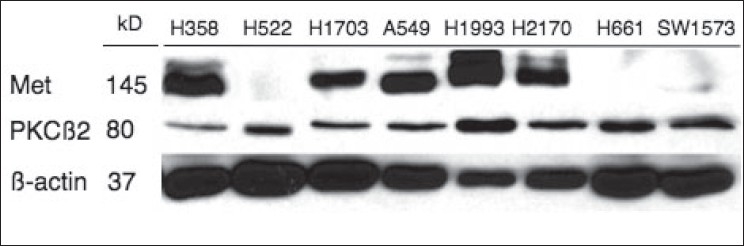
Expression of PKCß and MET in NSCLC cell lines. Immunoblotting performed as materials and methods. Represented are 8 cell lines of various histologies – adenocarcinoma (H522, H1703, H1993), bronchoalveolar carcinoma (SW1573, H358), adenosquamous carcinoma (A549), squamous cell carcinoma (H2170) and large cell carcinoma (H661). ß-actin was used as a loading control

In order to further investigate the potential therapeutic implications of concomitant PKCß and MET inhibition, eight NSCLC cell lines were evaluated for protein expression by immunoblotting. MET expression was robustly observed in 5/8 cell lines (H358, H1703, A549, H1993, H2170), while PKCß expression was seen in all 8 cell lines [Figure 2].

### NSCLC Cell Growth Inhibition with Enzastaurin and SU11274 Treatment:

**Figure 3:**
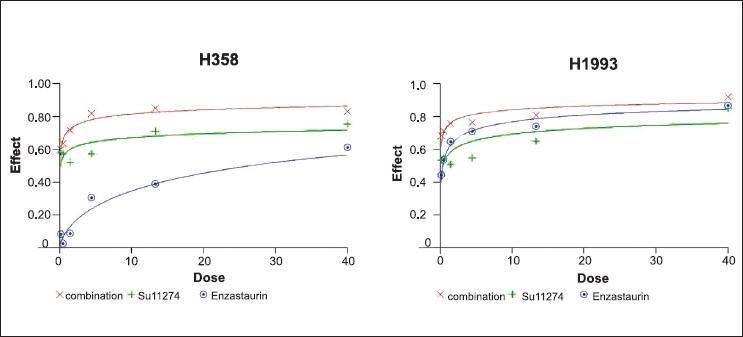
Combination of MET and PKCß inhibition in NSCLC cell lines. Dose-effect curves of H358 and H1993 cell lines treated with SU11274, enzastaurin or combination for 72 hours. Ordinates represent the fraction of (1 - cell mass) relative to control cell mass treated with diluent only. Each data point is an average of three wells. Curves are a function of the log (inhibitor [μM}) with a fixed slope (Hill slope). Represented are enzastaurin (blue), SU11274 (green) and combination (red).

Cell growth inhibition with various drugs was evaluated in H1993 and H358 cell lines. After treating these cells for 72 hours with SU11274, enzastaurin, or both drugs in combination in increasing concentrations, it was observed that the combination was synergistic in causing significant effects on cell proliferation in both cell lines. Combinatorial indices for H358 cells at ED50 was 0.32, and for H1993 was 0.09, therefore proving significant synergistic effect [Figure 3].

### Effects on Cell Signal Transduction with Enzastaurin and SU11274 Treatment:

**Figure 4:**
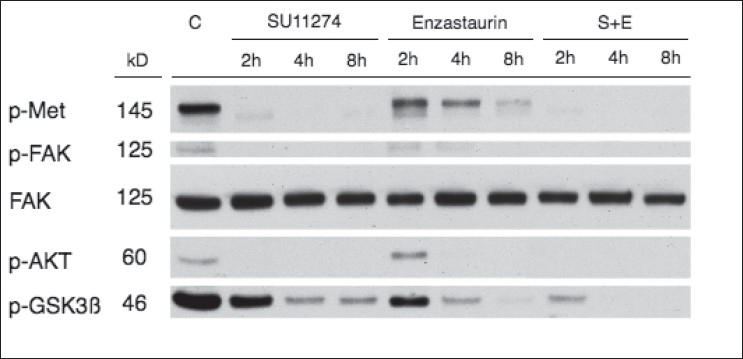
Biochemical effects of inhibition of MET and/or PKCß in NSCLC. Immunoblotting of H1993 cell lines treated with SU11274 (2.5 μM), enzastaurin (2.5 μM) or the combination (S+E) for different time points. Shown are p-MET, p-FAK, FAK, p-AKT and p-GSK3ß (see methods for specific antibodies used). Control lysates received treatment with diluent. FAK expression was used as loading control.

In order to investigate the molecular effects of treatment with SU11274 and/or enzastaurin, immunoblotting with phospho-specific antibodies was carried out in H1993 cells treated with these drugs for the time periods shown [Figure 4]. Treatment of H1993 cells with SU11274 completely inhibited phosphorylation of MET, FAK, and AKT within two hours after the initiation of treatment; however, an incomplete reduction in phosphorylation levels of GSK3ß was observed. Treatment with enzastaurin revealed mild reductions in phosphorylation of MET and incomplete abrogation of FAK, AKT, and GSK3ß phosphorylation. When both SU1174 and enzastaurin were used concomitantly, complete elimination of phosphorylation of MET, FAK, and AKT was observed, with near-total abrogation of GSK3ß phosphorylation [Figure 4].

## DISCUSSION

There have been multiple examples of the utilization of a combination of targeted agents in lung cancer. Unfortunately, the accumulated experience has been largely unsuccessful in the treatment of this disease. Our work presents a combination that might prove to be biologically relevant, and perhaps have a reasonable toxicity profile to be investigated in the clinical setting. Herein, we have identified that MET and PKCß are expressed in lung cancer, and their inhibition can be synergistic. Interestingly, also, metastasis is common in lung cancer, and lymph node metastasis correlated with both MET and PKCß expression.

For nearly two decades, MET has been known to be commonly aberrant in lung cancer, whether overexpressed,[21,28,29] amplified,[30–34] mutated,[35–37] or alternatively spliced,[34,38] and it therefore remains the focus of intense investigation. Binding of the MET receptor by its ligand HGF leads to receptor dimerization and activation of its intrinsic tyrosine kinase, followed by internalization into clathrin-coated vesicles, delivery to sorting endosomes, and degradation via the lysosomal pathway. Phosphorylation of MET at Y1230, Y1234, and Y1235 in the activation loop of the tyrosine kinase domain correlates with increased tyrosine kinase activity. In our inhibition experiments, phosphorylation of MET considerably decreased with SU11274 treatment.

MET activation can lead to autophosphorylation or phosphorylation of downstream intermediates and activation of signaling pathways. As an example, in small cell lung cancer (SCLC), activation of MET with HGF leads to phosphorylation/activation of several pathways involving cell proliferation/survival (ERK1/2, AKT), cell cycle (RB), and cytoskeletal proteins (paxillin, FAK).[39]

MET has also been shown to signal synergistically with EGFR as part of a broad signaling network that cooperatively drives activation of these downstream pathways.[22,40] Indeed, concomitant inhibition of MET and EGFR in erlotinib-resistant cells harboring the T790M mutation significantly increases lung cancer cytotoxicity above MET-targeted therapy alone in both *in vitro* and *in vivo* settings.[21]

Inhibition of PKCß with enzastaurin has been recently studied in thoracic malignancies. Our previous work has demonstrated the *in vitro* effect of enzastaurin against malignant pleural mesothelioma,[41] and its synergistic activity when combined with cisplatin. In NSCLC, *in vitro* activity of enzastaurin and pemetrexed, a commonly used antifolate compound, has also revealed synergistic activity against SW1573 and A549 cell lines. Multiple biochemical pathways were shown to be affected, such as cell cycle control, apoptosis, and angiogenesis.[42] In another recent publication, enzastaurin has been shown to be able to reverse acquired resistance to gefitinib, an EGFR small molecule inhibitor; while this study evaluated cell lines that are not NSCLC (colon cancer and prostate cancer), the mechanism observed may likely be observed in NSCLC.[43] The efficacy of combination enzastaurin and other cytotoxic agents in NSCLC might be dependent upon the schedule by which these drugs are delivered. Morgillo et al, have investigated the antiproliferative effects of enzastaurin with two commonly used drugs in the treatment of NSCLC, gemcitabine and pemetrexed. A synergistic effect was only observed when enzastaurin treatment was undertaken following the delivery of either gemcitabine or pemetrexed, while an antagonistic effect was observed if enzastaurin treatment preceded the cytotoxic agents.[44] A phase II clinical trial in which enzastaurin was used as a single agent as second- or third-line against NSCLC did not meet its primary end-point (an increase in progression-free survival of 20%); however, 13% of patients treated had progression-free survival greater than six months, signaling that perhaps a subset of patients with NSCLC might benefit from this drug.[45]

We have shown here that MET and PKCß tend to be coexpressed in NSCLC cell lines and tissues and that simultaneous inhibition of MET and PKCß significantly decreased cell proliferation in *in vitro* assays. This finding was associated with decrease in activation of downstream effectors such as GSK3ß, AKT and FAK. These data suggest that concomitant inhibition of MET and PKCß may be an effective treatment strategy for NSCLC, especially for those patients whose tumors have developed prior tyrosine kinase resistance. These findings warrant further investigation in vivo to determine whether such a dual inhibition strategy is effective in reducing tumor progression.

## AUTHOR'S PROFILE

**Dr. Ravi Salgia,** is a Professor of Medicine and the Director of the Thoracic Oncology Research Program at the University of Chicago Medical Center.
